# Inhibitory Effect of Tumor Suppressor p53 on Proinflammatory Chemokine Expression in Ovarian Cancer Cells by Reducing Proteasomal Degradation of IκB

**DOI:** 10.1371/journal.pone.0051116

**Published:** 2012-12-31

**Authors:** Deok-Soo Son, Syder M. Kabir, Yuan-Lin Dong, Eunsook Lee, Samuel E. Adunyah

**Affiliations:** 1 Department of Biochemistry and Cancer Biology, Meharry Medical College, Nashville, Tennessee, United State of America; 2 Department of Physiology, Meharry Medical College, Nashville, Tennessee, United State of America; Wayne State University School of Medicine, United States of America

## Abstract

Ovarian cancer, one of inflammation-associated cancers, is the fifth leading cause of cancer deaths among women. Inflammation in the tumor microenvironment is associated with peritoneal tumor dissemination and massive ascites, which contribute to high mortality in ovarian cancer. Tumor suppressor p53 is frequently deleted or mutated in aggressive and high-grade ovarian cancer, probably aggravating cancer progression and increasing mortality. We therefore investigated the influence of p53 on proinflammatory chemokines in ovarian cancer cells. A PCR array of the chemokine network revealed that ovarian cancer cells with low or mutated p53 expression expressed high levels of proinflammatory chemokines such as CXCL1, 2, 3 and 8. Transient transfection of p53 into p53-null ovarian cancer cells downregulated proinflammatory chemokines induced by tumor necrosis factor-α (TNF), a proinflammatory cytokine abundantly expressed in ovarian cancer. Furthermore, p53 restoration or stabilization blocked TNF-induced NF-κB promoter activity and reduced TNF-activated IκB. Restoration of p53 increased ubiquitination of IκB, resulting from concurrently reduced proteasome activity followed by stability of IκB. A ubiquitination PCR array on restoration of p53 did not reveal any significant change in expression except for Mdm2, indicating that the balance between p53 and Mdm2 is more important in regulating NF-κB signaling rather than the direct effect of p53 on ubiquitin-related genes or IκB kinases. In addition, nutlin-3, a specific inducer of p53 stabilization, inhibited proinflammatory chemokines by reducing TNF-activated IκB through p53 stabilization. Taken together, these results suggest that p53 inhibits proinflammatory chemokines in ovarian cancer cells by reducing proteasomal degradation of IκB. Thus, frequent loss or mutation of p53 may promote tumor progression by enhancing inflammation in the tumor microenvironment.

## Introduction

Ovarian cancer is the fifth leading cause of cancer deaths among women because it is typically asymptomatic and often diagnosed late until tumors have spread far beyond the ovaries [1]. Although the precise etiology remains unknown, increasing evidence indicates that ovarian cancer is associated with chronic inflammation [2]–[3]. Ovarian cancer tissue expresses high levels of CXCL1; similarly, serum levels of CXCL1 are higher in ovarian cancer patients than controls [4]–[5]. Advanced (less differentiated) ovarian cancer also overexpress CXCL8 in cyst fluids [6] and tumor cells [7]. Ovarian carcinoma ascitic fluid has also been found to contain high levels of CXCL8 [8]. In addition, paclitaxel-resistant ovarian cell lines express increased CXCL8 when compared with paclitaxel-sensitive cells [9]. Inflammatory reaction induces mainly proinflammatory chemokines such as CXCL1, 2 and 8 via NF-κB signaling in ovarian epithelial cancer cells [10]. Also proinflammatory tumor microenvironment is known to promote cancer progression. Therefore, together these factors likely contribute to the clinical features of ovarian cancer that cause high mortality, such as peritoneal tumor dissemination and massive ascites.

While the mechanism by which upregulation of proinflammatory chemokines in ovarian cancer remains unknown; a likely cause is activation of NF-κB resulting from loss of the tumor suppressor p53. Genetic alterations in p53 such as mutation and deletion are frequently observed in high-grade malignant ovarian cancer [11]. Accumulated evidence indicates that p53 represses NF-κB signaling through downregulation of IκB kinase (IKK) [12]–[14] or competition for transcriptional coactivators p300/CREB-binding protein (CBP) [15]–[17]. Interestingly, others have found that p53 promotes NF-κB activation [18]–[19]. Despite of controversial effects of p53 on NF-κB signaling, mutations or deletions of p53 can aggravate ovarian cancer progression based on the fact that mice deficient for p53 are prone to develop cancer [20]. We therefore hypothesize that functional loss of p53 in ovarian cancer can increase expression of proinflammatory chemokines by de-restricting NF-κB signaling.

In this study we restored p53 in ovarian cancer cells to determine its effects on proinflammatory chemokine expression in response to inflammatory stimuli. Further, we explored the mechanism by which this might occur by measuring degradation of IκB, proteasome activity, and expression of Mdm2, a negative regulator of p53 and an E3 ubiquitin ligase.

## Materials and Methods

### Reagents

Recombinant human TNF and human p53 DuoSet® IC ELISA kit were obtained from R&D Systems (Minneapolis, MN). Antibodies were purchased from the following vendors: p65, Mdm2 and β-actin from Santa Cruz Biotechnology (Santa Cruz, CA) and p53, p21, phosphorylated IκB, IκB, ubiquitin and IKK isoforms from Cell Signaling Technology (Beverly, MA). Lipofectamine 2000, TRIzol®, M-MLV, Taq DNA polymerase, and all liquid culture media were acquired from Invitrogen (Grand Island, NY). The PCR Array for customized human chemokines and ubiquitination pathway, PCR primers for CCL20, CXCL1, 2, 3, 8 and β-actin, and SYBR® Green Master Mix came from SABiosciences/Qiagen (Frederick, MD). Nutlin-3 was purchased from Cayman Chemical (Ann Arbor, MI). Chemiluminescent detection kits came from GE Healthcare (Piscataway, NJ). The p53 expression and pNF-κB-luc vectors came from BD Biosciences (Palo Alto, CA). The Luciferase Reporter Assay System and Proteasome Assay were obtained from Promega (Madison, WI).

### Cell lines and cell culture

The human ovarian cancer cell lines OVCAR-3, SKOV-3, CaOV-3 and TOV-21G were purchased from the American Type Culture Collection (Manassas, VA). A2780 and IGROV-1 ovarian cancer cell lines with p53 were kindly provided by Dr. Andrew Godwin (Fox Chase Cancer Center, Philadelphia, PA) [21] and Dr. Khabele (Vanderbilt University, Nashville, TN) [22], respectively. Human cells (approximately 5×10^4^ cells/ml) were cultured at 37°C in a water-saturated atmosphere of 95% air and 5% CO_2_ in 24- or 6-well plates with RPMI medium containing 10% FBS with penicillin (100 U/ml)/streptomycin (100 U/ml). The mouse ovarian surface epithelial cancer cell line (ID8) was kindly provided by Drs. Katherine Roby and Paul Terranova (University of Kansas Medical Center, Kansas City, KS) [23]. ID8 cells were cultured in Dulbecco's Modified Eagles Medium (DMEM) containing 4% FBS supplemented with penicillin/streptomycin. After overnight culture to allow cellular attachment to the plates, the medium was removed and fresh medium without FBS was added to remove the effects of serum.

### PCR array and real-time PCR

After isolating total RNA and eliminating genomic DNA, the RT reaction was performed at 42°C for 15 min followed by 94°C for 5 min. According to manufacturers' instructions, a real-time PCR reaction was performed using a Bio-Rad CFX96 (Hercules, CA) under the following two-step cycling program: 1 cycle at 95°C for 10 min, and 40 cycles at 95°C for 15 sec and at 60°C for 1 min. Data analysis was performed based on a Web-Based PCR Array Data Analysis protocol (http://pcrdataanalysis.sabiosciences.com/pcr/arrayanalysis.php) provided by SABiosciences in Qiagen (Frederick, MD).

### Transient transfection and luciferase assays

Human ovarian cancer cells at approximately 50% confluency in 6 or 24-well plates were washed once with fresh media without additives and were transiently transfected for 24 h at 37°C using Lipofectamine solution. Transfected cells were treated as outlined in Results and incubated for 6 h. After rinsing cells with ice-cold PBS and adding lysis buffer (Promega, Madison, WI), cell lysates were used for determination of luciferase activity using a microplate luminometer. Luciferase activity, expressed as relative light units, was normalized to measured protein levels.

### Western blot

Cell lysates were prepared, resolved on SDS-polyacrylamide gels, and transferred to nitrocellulose membranes according to established procedures as described previously [10]. Blocking of nonspecific proteins was performed by incubation of the membranes with 5% nonfat dry milk in Tris buffered saline Tween-20 for 2 h at room temperature. Blots were incubated with primary antibodies at 1∶1,000 dilution in blocking solution overnight at 4°C. The membranes were washed 3 times with TBST for 10 min and incubated for 1 h with horseradish peroxidase-conjugated secondary antibody at 1∶2,500 in 5% milk/TBST. The membranes were then rinsed 3 times with TBST for 10 min and the bands were visualized by enhanced chemiluminescence. After membrane stripping for 10 min with methanol containing 3% H_2_O_2_, β-actin was detected in order to serve as an internal loading control.

### Immunoprecipitation (IP) and immunoblotting (IB)

Cell lysates (1000 µg of total cellular protein/ml) were prepared and incubated with 10 µl primary antibody for 2 h at 4°C. Then 20 µl of Protein A/G PLUS-Agarose was added and cell lysates were incubated at 4°C on a rocker overnight. Pellets were collected by centrifuging at approximately 1,000×*g* for 5 min at 4°C. After carefully discarding supernatant, the pellets were washed 3 times with RIPA buffer by centrifuging at approximately 1,000×*g* for 5 min at 4°C. After the final wash, the pellets were resuspended in 40 µl of 2× electrophoresis sample buffer and boiled for 2 min. Electrophoresis and immunoblotting was performed as described in Western Blot.

### Enzyme-linked immunosorbent assay (ELISA)

Human p53 activity was measured by human p53 ELISA kit (R&D Systems, Minneapolis, MN) according to manufacturer's instructions. The optical density of each well was determined, using a microplate reader set to 450 nm with wavelength correction at 570 nm.

### Cell-based proteasome assay

After transient transfection of p53 in 24-well plates, cells were incubated with the Promega Proteasome-Glo™ Chymotrypsin-Like Cell-Based Assay Reagent (Promega, Madison, WI) for 10 min according to manufacturer's instructions. Luciferase activity was determined using a microplate luminometer.

### Statistics

Data were analyzed by the paired Student's *t*-test and one-way analysis of variance (ANOVA) as appropriate. If statistical significance (p≤0.05) was determined by ANOVA, the data were further analyzed by Tukey's pairwise comparison to detect specific differences between treatments.

## Results

### Signature of chemokine network and p53 in ovarian cancer cells

**Figure 1:**
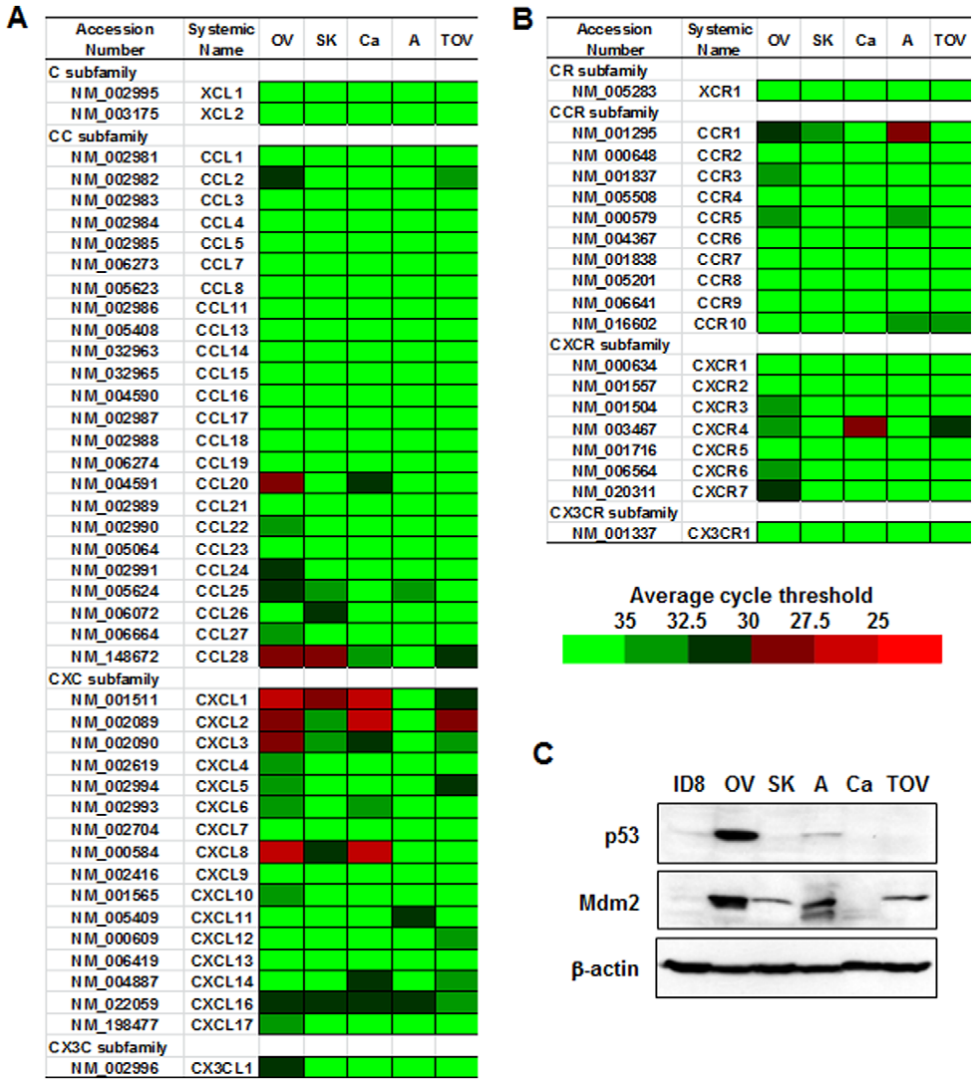
Ovarian cancer cell lines tend to express high levels of proinflammatory chemokines and have low p53 activity. (A) Signature of chemokine ligands and (B) chemokine receptors in human ovarian cancer cell lines. After isolating total RNA from each cell line, PCR array was performed using a customized PCR array plate containing complementary sequences for human chemokine genes. Different colors indicate average cycle threshold with expression ranges from >35 to <25. (C) Protein expression of p53 and Mdm2 in ovarian cancer cell lines. Whole cell lysates were prepared and Western blot was carried out using antibodies specific to p53, Mdm2 and β-actin as loading control. Experiments were performed in duplicate and a representative result is shown. OV, OVCAR-3 cells; SK, SKOV-3 cells; A, A2780 cells; Ca, CaOV-3 cells; TOV, TOV-21G cells.

As a preliminary test of whether loss of p53 in ovarian cancer cells increases expression of proinflammatory cytokines, we examined expression of chemokines and p53 in the established ovarian cancer cell lines ID8, OVCAR-3, SKOV-3, A2780, CaOV-3 and TOV-21G. We used a PCR array for the chemokine network, which includes genes for chemokines and chemokine receptors, and determined average cycle thresholds from <25 cycle to >35 cycles. The results revealed high expression of chemokines in the following ovarian cancer cell lines: CCL20 and 28, CXCL1, 2, 3 and 8 in OVCAR-3 cells; CCL28 and CXCL1 in SKOV-3 cells; CXCL1, 2 and 8 in CaOV-3 cells; and CXCL2 in TOV-21G cells (Figure 1A). Interestingly A2780 cells were did not express or expressed low levels of almost all chemokines when compared with other ovarian cancer cells. Further, CCR1 and CXCR4 were highly expressed in A2780 and CaOV-3 cells, respectively (Figure 1B). Even p53 wild-type IGROV-1 cells expressed highly CXCL3, CXCL14, CCR10 and CXCR4 (Figure S1A).

In addition, we analyzed protein levels of p53 and Mdm2 in the same cell lines. OVCAR-3 cells highly expressed p53 protein, while ID8 and A2780 cells expressed relatively lower levels of p53. Clearly, SKOV-3, CaOV-3, and TOV-21G cells did not express p53. We also examined expression of Mdm2, a negative regulator of p53. Mdm2 was highly expressed in A2780 and OVCAR-3 cells in contrast to no expression in ID8 and CaOV-3 cells (Figure 1C). SKOV-3 and TOV-21G cells expressed Mdm2 in spite of the loss of p53 (Figure 1C). These results suggest that p53 is either absent or expressed at very low levels in most cell lines studied.

### Effect of p53 on TNF-induced chemokines

**Figure 2:**
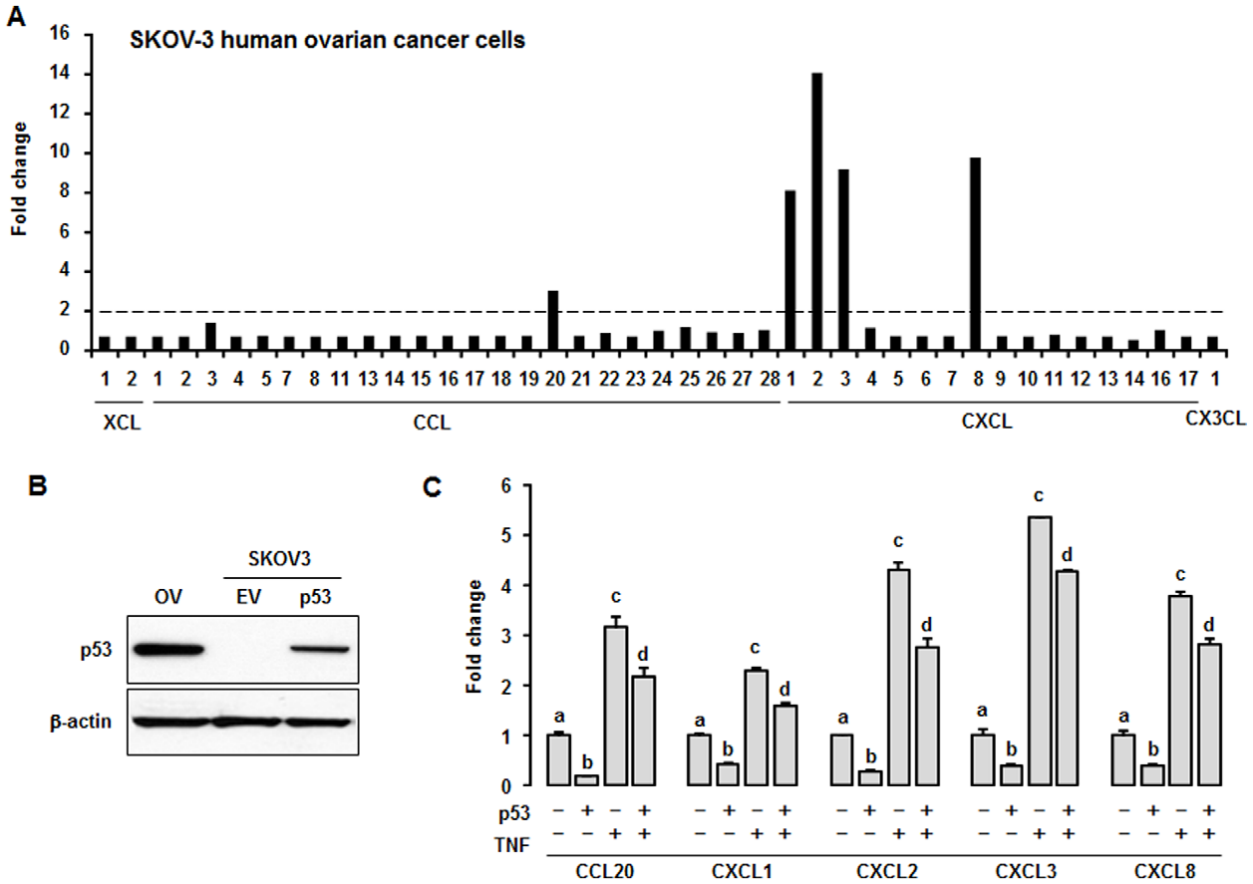
Overexpression of p53 downregulates TNF-induced chemokines. (A) TNF-induced chemokines in SKOV-3 cells. After isolating total RNA, PCR array was performed using a human chemokine PCR array plate. Dotted line indicates 2-fold increase; chemokines with a greater than 2-fold increase are recognized as TNF-induced chemokines. (B) Confirmation of p53 protein expression after transient transfection in SKOV-3 cells. After transfection of empty vector (EM) and p53 expression vector (p53), whole cell lysates were prepared and p53 expression was confirmed by Western blot. β-actin is used as a loading control. (C) Effect of p53 on TNF-induced chemokines. After overnight transfection of vectors, cells were treated with TNF (10 ng/ml) for 1 h and qRT-PCR was carried out using primers for CCL2, CXCL1, 2, 3 and 8. β-actin serves as normalization control. Different letters indicate significant differences (P≤0.05) within each chemokine group (ANOVA and Tukey's pairwise comparisons). Experiments were performed in triplicate and all data are shown as mean ± SE.

TNF is a proinflammatory cytokine abundantly expressed in ovarian cancer [24]. We selected SKOV-3 cells as a p53-null model ovarian cancer cell to identify responsive chemokines to TNF. TNF specifically induced proinflammatory chemokines such as CCL20, CXCL1, 2, 3 and 8 (Figure 2A). Furthermore, we transiently transfected p53 into SKOV-3 cells to measure the influence of p53 on TNF-induced chemokines (Figure 2B). Restoration of p53 into SKOV-3 cells led to downregulation of proinflammatory chemokines at both basal and TNF-induced levels (Figure 2C). These results suggest that functional loss of p53 in ovarian cancer can increase expression of proinflammatory chemokines, resulting to inflammation in the tumor microenvironment.

### Effect of p53 on NF-κB promoter activity and TNF-activated IκB

**Figure 3:**
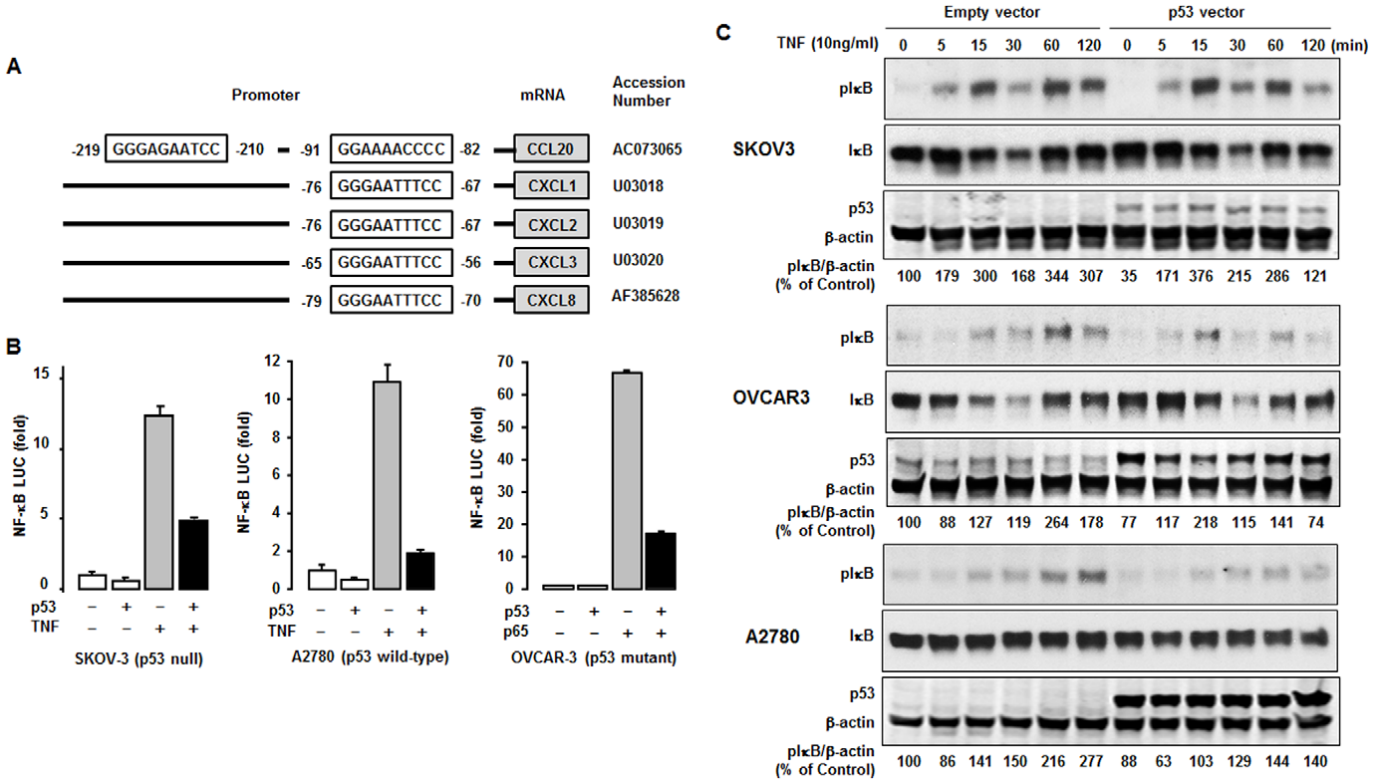
Overexpression of p53 inhibits NF-κB activity. (A) Nucleotide sequences of promoters for TNF-induced chemokines such as CCL20, CXCL1, 2, 3 and 8. These chemokine promoters contain one NF-κB site at the proximal region, except for CCL20, which has two NF-κB sites at the distal and proximal region. (B) Effect of p53 on NF-κB luciferase activity. After transfection of vectors or cotransfection with p65, cells were treated with TNF (10 ng/ml) for 4 h. (C) Effect of p53 on TNF-activated IκB. After transfection of empty vector or p53 in SKOV-3 (p53 null), OVCAR-3 (p53 mutant) and A2780 (p53 wild-type), cells were treated with TNF (10 ng/ml) for indicated times. β-actin serves as loading control. Experiments were performed in duplicate and a representative result is shown; numbers below are relative density values.

TNF-induced chemokines such as CCL20, CXCL1, 2, 3 and 8 contain proximal κB sites on their promoters (Figure 3A). We therefore employed an NF-κB-driven luciferase reporter vector to confirm involvement of NF-κB in the inhibitory effect of p53 on TNF and p65 (a subunit of NF-κB) induction of chemokine expression. We selected three cell lines that vary in terms of p53 expression: SKOV-3 (p53 null), A2780 (p53 wild-type) and OVCAR-3 (p53 mutant) cells [25]–[26]. Regardless of the status of p53, p53 restoration significantly reduced TNF- and p65-induced NF-κB promoter activity (Figure 3B). These results suggest that loss of p53 in ovarian cancer can increase proinflammatory chemokines by enhancing NF-κB promoter activity in response to inflammatory reaction.

We further explored whether p53 affects TNF-induced activation of IκB in these transiently transfected cells. Although p53 restoration had no appreciable effect on TNF-activated IκB at 5 through 30 min, it reduced activation of IκB at 1 to 2 h. In spite of varying expression of p53, this reduced effect at late time points was similar in all cell lines tested (Figure 3C). Reduced activation of IκB at late time points suggest that p53 is likely to affect events related with NF-κB complex (IκB-p65/p52) rather than an upstream of NF-κB in ovarian cancer cells.

### Involvement of p53 in IκB degradation

**Figure 4:**
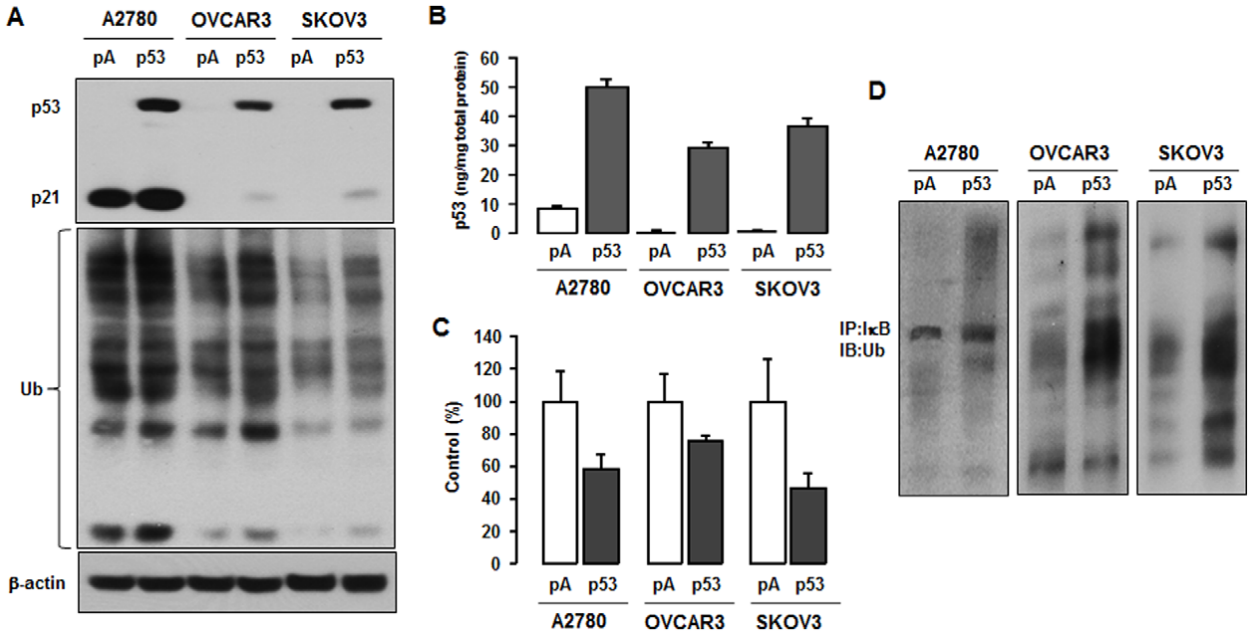
Restoration of p53 delays IκB degradation. (A) Accumulated effect of p53 on ubiquitylated proteins. After transient transfection of p53 in A2780, OVCAR-3 and SKOV-3 cells, whole cell lysates were prepared and Western blot was carried out using antibodies specific to ubiquitin, p21, IκB, p53 and β-actin (as loading control). Experiments were performed in duplicate and a representative result is shown. (B) Confirmation of p53 activity after transient transfection of p53. ELISA was performed in triplicate and data are shown as mean ± SE. Dark gray bars indicate significance (p<0.05, paired Student's *t*-test) within each cell line. (C) The effect of p53 on proteasome activity. Assays were performed in triplicate and data are shown as mean ± SE. Dark gray bars indicate significance (p<0.05, paired Student's *t*-test) within each cell line. (D) Effects of p53 on ubiquitination of IκB. Immunoprecipitated IκB was immunoblotted using ubiquitin antibody. Experiments were performed in duplicate and a representative result is shown.

One mechanism by which IκB is regulated is by proteasomal degradation after ubiquitination. We investigated whether this mechanism was involved in p53's ability to reduce IκB activation. After transient transfection of p53, we confirmed the function of p53 by examining whether it increased expression of p21, a potent cyclin-dependent kinase inhibitor tightly controlled by p53. A2780 cells constitutively expressed p21 whereas OVCAR-3 and SKOV-3 cells did not express p21. Overexpression of p53 increased p21 protein level in all cell lines (Figure 4A). In addition, p53 significantly enhanced accumulation of ubiquitinated proteins in the whole cell lysates (Figure 4A), probably by disrupting the degradation system. ELISA assay revealed that restoration of p53 increased p53 activity in all cell lines (Figure 4B). The p53 wild-type A2780 cells expressed basal p53 activity whereas p53 mutant OVCAR-3 and p53 null SKOV-3 cells showed no p53 activity (Figure 4B). To determine the cause of this accumulation, we measured proteasome activity and found it was reduced by overexpression of p53 in all cell lines tested (Figure 4C). Moreover, after immunoprecipitation of IκB, we confirmed that p53 increased ubiquitination of IκB (Figure 4D). Accumulating ubiquitinated IκB by p53 suggests that p53 blocks degradation of IκB by reducing proteasome activity in ovarian cancer cells.

### Effect of p53 on ubiquitin-related genes

The effect of p53 on ubiquitin-related genes is shown in table 1. Though decreased proteasome activity would explain the increase in ubiquitinated proteins, increased expression of ubiquitin-related genes could also contribute. Therefore, using a PCR array we investigated whether p53 directly affects ubiquitin-related genes such as ubiquitin-activating enzymes (E1), ubiquitin-conjugating enzymes (E2) and ubiquitin-protein ligases (E3). A PCR array for ubiquitination genes revealed that overexpression of p53 had no direct effects on E1 or E2 except an approximately 2-fold decrease of UBE2E2 in SKOV-3 cells. Although in A2780 and OVCAR-3 cells, p53 overexpression caused a 2–3 fold increase in the ubiquitin ligases CUL9 and RNF148 expression, respectively, PCR array revealed that these genes were expressed at low levels. Interestingly the p53 specifically increased expression of Mdm2, a negative regulator of the p53 tumor suppressor and an E3 ubiquitin ligase, in all cell lines tested. Notably the increasing effect of p53 on Mdm2 was 1.53 fold in A2780 cells, 5.41-fold in OVCAR-3 and 3.13-fold in SKOV-3 cells. Mdm2 as a p53-inducible gene in ubiquitin-related genes is likely to be involved in repressing p53 activity which attenuates NF-κB signaling through an autoregulatory negative feedback loop.

**Table 1:** Effect of p53 on ubiquitin-activating enzymes (E1), ubiquitin-conjugating enzymes (E2) and ubiquitin-protein ligases (E3) obtained from comparison between empty vector and p53 vector transfected ovarian cancer cells.

Ubiquitin-activating enzymes (E1)								
Symbol	Access No.	Ap53	Level	OVp53	Level	SKp53	Level	Description
ATG7	NM_006395	1.07	H	1.16	H	0.78	H	ATG7 autophagy related 7 homolog (S. cerevisiae)
NAE1	NM_003905	0.96	H	1.17	H	1.11	H	NEDD8 activating enzyme E1 subunit 1
SAE1	NM_005500	1.05	H	1.05	H	0.83	H	SUMO1 activating enzyme subunit 1
UBA1	NM_003334	0.84	H	1.08	H	0.80	H	Ubiquitin-like modifier activating enzyme 1
UBA2	NM_005499	0.86	H	1.01	H	1.17	H	Ubiquitin-like modifier activating enzyme 2
UBA3	NM_003968	0.96	H	1.00	H	0.77	H	Ubiquitin-like modifier activating enzyme 3
UBA5	NM_198329	1.17	H	1.08	H	0.87	H	Ubiquitin-like modifier activating enzyme 5
UBA6	NM_018227	0.88	H	1.18	H	1.09	H	Ubiquitin-like modifier activating enzyme 6

Ubiquitin-conjugating enzymes (E2)								
Symbol	Access No.	Ap53	Level	OVp53	Level	SKp53	Level	Description
ARIH1	NM_005744	1.07	H	1.17	H	0.90	H	Ariadne homolog, ubiquitin-conjugating enzyme E2 binding protein
CDC34	NM_004359	1.42	H	1.10	H	0.94	H	Cell division cycle 34 homolog (S. cerevisiae)
NEDD8	NM_006156	0.90	H	0.98	H	0.87	H	Neural precursor cell expressed, developmentally down-regulated 8
TMEM189	NM_199129	0.88	H	0.97	H	1.15	H	Transmembrane protein 189
UBE2A	NM_003336	0.82	H	1.08	H	0.81	H	Ubiquitin-conjugating enzyme E2A (RAD6 homolog)
UBE2B	NM_003337	0.91	H	1.05	H	1.12	H	Ubiquitin-conjugating enzyme E2B (RAD6 homolog)
UBE2C	NM_181803	0.86	H	1.01	H	1.23	H	Ubiquitin-conjugating enzyme E2C
UBE2D1	NM_003338	0.95	H	1.11	H	0.94	H	Ubiquitin-conjugating enzyme E2D 1 (UBC4/5 homolog, yeast)
UBE2D2	NM_181838	0.90	H	1.00	H	0.92	H	Ubiquitin-conjugating enzyme E2D 2 (UBC4/5 homolog, yeast)
UBE2D3	NM_181893	0.92	H	1.01	H	0.89	H	Ubiquitin-conjugating enzyme E2D 3 (UBC4/5 homolog, yeast)
UBE2E1	NM_182666	1.03	H	0.96	H	0.76	H	Ubiquitin-conjugating enzyme E2E 1 (UBC4/5 homolog, yeast)
UBE2E2	NM_152653	1.06	H	0.98	H	**0.44**	H	Ubiquitin-conjugating enzyme E2E 2 (UBC4/5 homolog, yeast)
UBE2E3	NM_006357	0.96	H	0.76	H	0.89	H	Ubiquitin-conjugating enzyme E2E 3 (UBC4/5 homolog, yeast)
UBE2G1	NM_003342	1.15	H	0.91	H	1.46	N/D	Ubiquitin-conjugating enzyme E2G 1 (UBC7 homolog, yeast)
UBE2G2	NM_182688	1.11	H	1.14	H	0.83	H	Ubiquitin-conjugating enzyme E2G 2 (UBC7 homolog, yeast)
UBE2H	NM_182697	1.03	H	0.95	H	0.81	H	Ubiquitin-conjugating enzyme E2H (UBC8 homolog, yeast)
UBE2I	NM_003345	1.05	H	1.01	H	0.92	H	Ubiquitin-conjugating enzyme E2I (UBC9 homolog, yeast)
UBE2J1	NM_016021	1.05	H	1.09	H	0.72	H	Ubiquitin-conjugating enzyme E2, J1 (UBC6 homolog, yeast)
UBE2J2	NM_194458	1.27	H	0.98	H	0.78	H	Ubiquitin-conjugating enzyme E2, J2 (UBC6 homolog, yeast)
UBE2K	NM_005339	0.90	H	1.11	H	0.96	H	Ubiquitin-conjugating enzyme E2K (UBC1 homolog, yeast)
UBE2L3	NM_003347	0.98	H	0.98	H	1.00	H	Ubiquitin-conjugating enzyme E2L 3
UBE2M	NM_003969	1.39	H	1.02	H	0.73	H	Ubiquitin-conjugating enzyme E2M (UBC12 homolog, yeast)
UBE2N	NM_003348	1.22	H	1.14	H	0.89	H	Ubiquitin-conjugating enzyme E2N (UBC13 homolog, yeast)
UBE2Q1	NM_017582	1.09	H	1.17	H	0.72	H	Ubiquitin-conjugating enzyme E2Q family member 1
UBE2R2	NM_017811	0.87	H	1.03	H	0.75	H	Ubiquitin-conjugating enzyme E2R 2
UBE2S	NM_014501	1.24	H	1.19	H	0.74	H	Ubiquitin-conjugating enzyme E2S
UBE2T	NM_014176	1.08	H	1.05	H	1.17	H	Ubiquitin-conjugating enzyme E2T (putative)
UBE2W	NM_018299	0.92	H	1.05	H	0.92	H	Ubiquitin-conjugating enzyme E2W (putative)
UBE2Z	NM_023079	1.09	H	0.93	H	0.78	H	Ubiquitin-conjugating enzyme E2Z
UBE4B	NM_006048	0.79	H	0.77	H	0.92	H	Ubiquitination factor E4B (UFD2 homolog, yeast)

Ubiquitin-ligase enzymes (E3)								
Symbol	Access No.	Ap53	Level	OVp53	Level	SKp53	Level	Description
ANAPC11	NM_001002244	0.77	H	0.98	H	1.01	H	Anaphase promoting complex subunit 11
ANAPC2	NM_013366	1.34	H	1.27	H	0.63	H	Anaphase promoting complex subunit 2
BTRC	NM_033637	1.12	H	0.91	H	0.79	H	Beta-transducin repeat containing
CUL1	NM_003592	0.97	H	1.05	H	1.02	H	Cullin 1
CUL2	NM_003591	0.84	H	1.15	H	1.00	H	Cullin 2
CUL3	NM_003590	0.92	L	1.25	L	0.90	L	Cullin 3
CUL4A	NM_003589	0.85	H	1.05	H	0.91	H	Cullin 4A
CUL4B	NM_003588	0.84	H	1.06	H	0.73	H	Cullin 4B
CUL5	NM_003478	0.89	H	0.96	H	1.18	H	Cullin 5
CUL7	NM_014780	1.46	H	1.00	H	0.70	H	Cullin 7
CUL9	NM_015089	**2.76**	L	1.22	H	0.81	H	Cullin 9
DZIP3	NM_014648	1.02	H	1.01	H	0.78	H	DAZ interacting protein 3, zinc finger
FBXO3	NM_012175	0.94	H	0.96	H	0.80	H	F-box protein 3
FBXO31	NM_024735	1.23	H	1.18	H	0.81	H	F-box protein 31
FBXO4	NM_012176	1.05	H	1.14	H	0.89	H	F-box protein 4
FBXW10	NM_031456	1.39	L	0.80	L	0.64	L	F-box and WD repeat domain containing 10
FBXW9	NM_032301	1.12	H	1.13	H	0.83	H	F-box and WD repeat domain containing 9
HECW1	NM_015052	1.38	N/D	1.51	L	0.71	L	HECT, C2 and WW domain containing E3 ubiquitin protein ligase 1
HECW2	NM_020760	1.05	H	0.94	H	0.80	H	HECT, C2 and WW domain containing E3 ubiquitin protein ligase 2
HERC5	NM_016323	1.24	H	1.87	H	1.90	H	Hect domain and RLD 5
HUWE1	NM_031407	1.03	H	1.08	H	0.60	H	HECT, UBA and WWE domain containing 1
MARCH5	NM_017824	1.24	H	1.20	H	0.95	H	Membrane-associated ring finger (C3HC4) 5
**MDM2**	NM_002392	1.53	H	**5.41**	H	**3.13**	H	Mdm2 p53 binding protein homolog (mouse)
MIB1	NM_020774	0.97	H	1.03	H	0.71	H	Mindbomb homolog 1 (Drosophila)
MUL1	NM_024544	1.11	H	1.04	H	0.73	H	Mitochondrial E3 ubiquitin ligase 1
NEDD8	NM_006156	0.90	H	0.98	H	0.87	H	Neural precursor cell expressed, developmentally down-regulated 8
PARK2	NM_004562	1.09	L	0.57	L	1.05	H	Parkinson disease (autosomal recessive, juvenile) 2, parkin
RFWD2	NM_022457	1.12	H	0.99	H	0.80	H	Ring finger and WD repeat domain 2
RNF123	NM_022064	1.53	H	1.00	H	0.81	H	Ring finger protein 123
RNF148	NM_198085	1.38	N/D	**2.21**	L	0.81	L	Ring finger protein 148
SKP1	NM_006930	0.85	H	0.98	H	1.00	H	S-phase kinase-associated protein 1
SKP2	NM_005983	1.02	H	0.98	H	0.82	H	S-phase kinase-associated protein 2 (p45)
SMURF1	NM_020429	1.11	H	1.08	H	0.91	H	SMAD specific E3 ubiquitin protein ligase 1
SMURF2	NM_022739	0.93	H	1.65	H	1.17	H	SMAD specific E3 ubiquitin protein ligase 2
STUB1	NM_005861	1.35	H	0.92	H	0.87	H	STIP1 homology and U-Box containing protein 1
SYVN1	NM_172230	1.00	H	1.32	H	0.71	H	Synovial apoptosis inhibitor 1, synoviolin
UBR1	NM_174916	1.19	H	1.24	H	1.23	H	Ubiquitin protein ligase E3 component n-recognin 1
UBR2	NM_015255	0.80	H	1.05	H	1.05	H	Ubiquitin protein ligase E3 component n-recognin 2
VHL	NM_000551	1.05	H	1.03	H	0.89	H	Von Hippel-Lindau tumor suppressor
WWP1	NM_007013	1.26	H	1.05	H	0.75	H	WW domain containing E3 ubiquitin protein ligase 1

Fold changes were compared with Control after normalization of housekeeping genes such as actin and glyceraldehyde-3-phosphate dehydrogenase. H: high level (<30 cycles), L: low levels (30–35 cycles) and N/D (>35 cycles): not determined.

### Effect of p53 on Mdm2 expression, binding with NF-κB components and IKK isoforms

**Figure 5:**
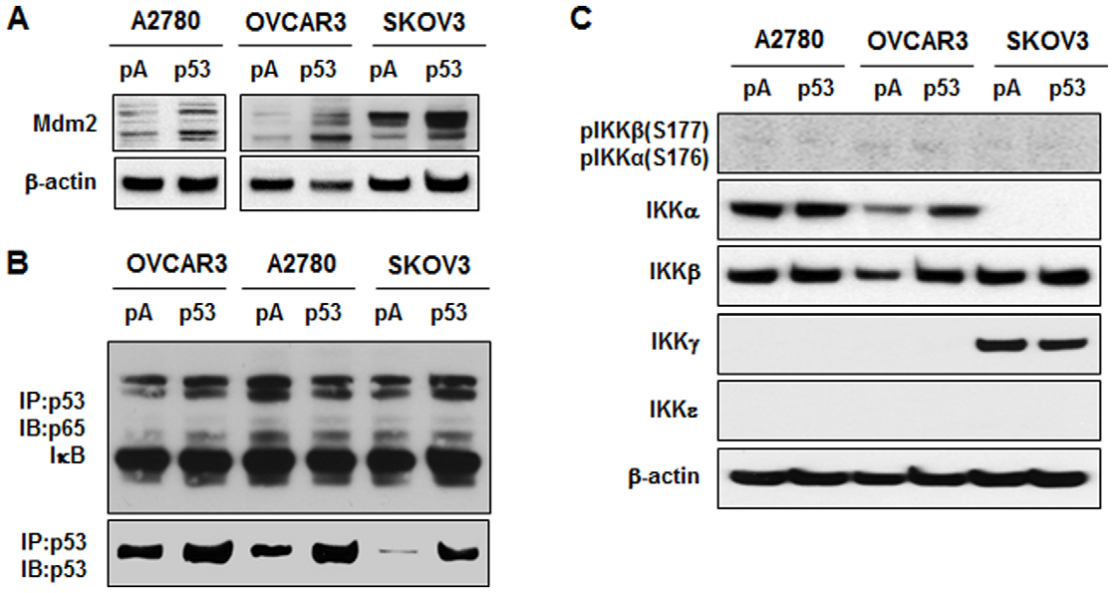
Overexpressed p53 induces Mdm2 whereas does not affect to the binding of NF-κB components and any IKK isoform. (A) Effect of p53 on Mdm2 expression. After transient transfection of p53 in A2780, OVCAR-3 and SKOV-3 cells, whole cell lysates were prepared and Western blot was carried out using antibodies specific to Mdm2; and β-actin served as loading control. (B) Effects of p53 expression on p53 binding to p65 and IκB. After transient transfection of p53, immunoprecipitated (IP) p53 was immunoblotted (IB) using p65 or IκB antibody. (C) Effect of p53 on expression of various IKK isoforms. After transient transfection of p53, whole cell lysates were prepared and Western blot was carried out using antibodies specific to IKKα, IKKβ, IKKγ, IKKε; β-actin served as loading control. Experiments were performed in duplicate and a representative result is shown.

Based on p53-induced Mdm2 mRNA indicated by PCR array (Table 1), we next confirmed the increased expression of Mdm2 protein by p53 in A2780, OVCAR-3 and SKOV-3 cells by Western blot (Figure 5A). Because IκBα is known to bind to p53 *in vitro* [27] and repress p53-dependent effects [28], overexpression of p53 could decrease NF-κB signaling by binding to p65 and IκB. Immunoprecipitation of p53 revealed that restoration of p53 had no significant effect on p53 binding to either p65 or IκB (Figure 5B). Because loss of p53 is known to enhance activity of IKKα or IKKβ [12]–[13], we clarified whether p53 was involved in regulating expression of IKK. Though each cell line expressed a distinct combination of IKK isoforms (IKKβ was expressed in all cell lines tested, SKOV3 cells expressed IKKγ instead of IKKα, and IKKε was not expressed in any cell line), overexpression of p53 had no effect on IKK expression and phosphorylation in any cell line (Figure 5C). These results suggest that perhaps dominant effect of p53 over Mdm2 is more important for attenuation of NF-κB signaling rather than direct effects on upstreams or components of NF-κB.

### Effect of nutlin-3, a specific inducer of p53 stabilization, on TNF-induced chemokines

**Figure 6:**
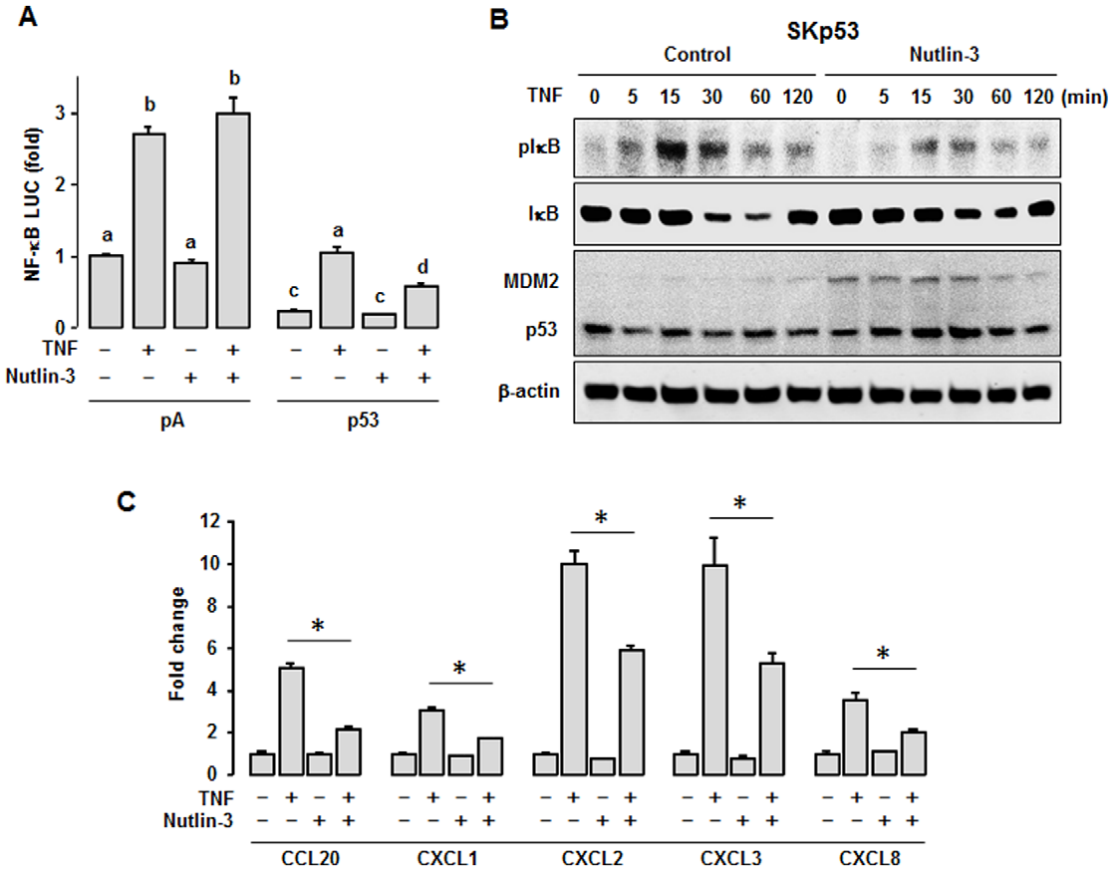
Nutlin-3, a p53 stabilizer, downregulates TNF-induced chemokines. (A) Effect of nutlin-3 on NF-κB luciferase activity. After transfection of vectors or cotransfection with p53, cells were pretreated with nutlin-3 (10 µM) for 24 h followed by TNF (10 ng/ml) for 4 h. Different letters indicate significant differences (P≤0.05) within each group (ANOVA and Tukey's pairwise comparisons). Experiments were performed in triplicate and all data are shown as mean ± SE. (B) Effect of nutlin-3 on TNF-activated IκB. After transfection of empty vector or p53 in SKOV-3 cells, cells were pretreated with nutlin-3 (10 µM) for 24 h followed by TNF (10 ng/ml) for indicated times. β-actin serves as loading control. Experiments were performed in duplicate and a representative result is shown. (C) Effect of nutlin-3 on TNF-induced chemokines. After overnight transfection of vectors, cells were pretreated with nutlin-3 (10 µM) for 24 h followed by TNF (10 ng/ml) for 1 h and qRT-PCR was carried out using primers for CCL2, CXCL1, 2, 3 and 8. β-actin serves as normalization control. Asterisk indicates significant differences (P≤0.05, paired Student's *t*-test) when compared to the presence of nutlin-3. Experiments were performed in triplicate and all data are shown as mean ± SE.

We employed nutlin-3 as a p53 stabilizer to confirm the inhibitory effect of p53 on TNF-induced chemokine expression. Nutlin-3 had no effect on TNF-induced NF-κB promoter activity in p53 null SKOV-3 cells but reduced its activity in p53-overexpressed SKOV-3 cells (Figure 6A). Continually we explored whether nutlin-3 affects TNF-activated IκB in p53-overexpressed SKOV-3 cells. Nutlin-3 reduced TNF-activated IκB by stabilizing p53 as indicated with augmentation of MDM2 (Figure 6B). Stabilization of p53 by nutlin-3 in p53-overexpressed SKOV-3 cells led to downregulation of TNF-induced proinflammatory chemokines (Figure 6C). These results confirm that functional loss of p53 activity in ovarian cancer can increase expression of proinflammatory chemokines, increasing inflammation burden in the tumor microenvironment. In addition, we employed p53 wild-type IGROV-1 cells to confirm in the inhibitory effect of nutlin-3 on TNF-activated IκB. Nutlin-3 decreased TNF-activated IκB by stabilizing p53 as indicated with augmentation of p21 and MDM2 in spite of low basal expression of p53 (Figure S1B). Though IGROV-1 cells expressed highly IKKα and IKKβ (IKKγ was lowly expressed and IKKε was not expressed), nutlin-3 had no effect on IKK phosphorylation (Figure S1C). Furthermore, we investigated differential effect of nutine-3 on TNF-activated IκB between p53 wild-type A2789 and p53 mutant OVCAR-3 cells. Nutlin-3 reduced TNF-activated IκB in p53 wild-type A2780 cells by stabilizing p53 followed by p21 and MDM2 augmentation (Figure S2A). On the other hand, nutlin-3 had no effect in p53 mutant OVCAR-3 cells as indicated with no change of p53 and MDM2 (Figure S2B). Other authors also demonstrated that nutlin-3 stabilized p53 with augmentation of p21 and MDM2 in other p53 wild-type cells but not in p53 mutant cells [29]-[30]. Nutlin-3 did not affect TNF-activated IKK in both cells (Figure S2). These results confirm that functional gain of p53 is more important for attenuation of NF-κB signaling rather than direct effects on upstreams of NF-κB.

## Discussion

This study demonstrates that p53 inhibits proinflammatory chemokines in ovarian cancer cells. This effect of p53 is likely resulted from attenuated NF-κB signaling by decreased proteasomal degradation of IκB and dominant effects of p53 over Mdm2.

This investigation was motivated by the fact that aggressive and high-grade ovarian cancer, an inflammation-associated cancer, frequently involves mutations or deletions of p53 [11]. Our first correlational experiment revealed that p53-null or mutated ovarian cancer cells highly expressed proinflammatory chemokines such as CCL20, CCL28, CXCL1, 2, 3 and 8 which agrees with our previous observations [10]. In addition, CCR1, CCR10 and CXCR4 were highly expressed in A2780 cells, CaOV-3 cells (Figure 1B) and IGROV-1 cells (Figure S1A). Because CCR1, CCR10 and CXCR4 are not specific receptors for the proinflammatory chemokines released by ovarian cancer cells, these receptors are likely to interact with chemokines released by other cell types such as immune cells and endothelial cells to the microenvironment.

TNF induced expression of CCL20, CXCL1, 2, 3 and 8, but not CCL28 which was highly expressed in SKOV-3 and OVCAR-3 cells (Figure 1A and 2A). The lack of induction of CCL28 by TNF in these ovarian cancer cells is in contrast to earlier report [31]-[32] that TNF and IL-1 increase expression of CCL28 in human keratinocyte cells and colon epithelium. In fact, CCL28 level remained unchangeable in all human ovarian cancer cells tested (data not shown). This difference indicates that chemokine network is likely to be differently regulated in various cell types.

Restoration of p53 into p53-null SKOV-3 cells attenuated expression of proinflammatory chemokines in both basal and TNF-induced conditions (Figure 2C). This result indicates that the loss or mutation of p53 observed frequently in ovarian cancer contributes to augmented proinflammatory chemokines in the tumor microenvironment, which is known to facilitate cancer progression.

Because the promoters of CCL20, CXCL1, 2, 3 and 8 contain κB sites (Fig. 3A) and proinflammatory chemokines are regulated by NF-κB signaling [10], p53 effects on chemokine expression likely involve changes in NF-κB signaling. In this study, restoration of p53 into ovarian cancer cells and nutlin-3, a p53 stabilizer, abolishes TNF-induced NF-κB promoter activity (Figure 3B and 6A). These results confirm and contrast with previous findings, as conflicting effects of p53 on NF-κB signaling have been found. Some evidence suggests that p53 activates the p65 subunit of NF-κB via the ribosomal S6 kinase 1 [18] and TNF triggers a transcriptionally active complex of p65 and p53 on κB response elements, indicating that mutated p53 rather than loss of p53 contributes to tumor progression [19]. On the other hand, accumulating evidence indicates a negative effect of p53 on NF-κB signaling, in agreement with our results. The p53 may repress NF-κB signaling by disrupting IKK expression as an upstream of NF-κB signaling. Although p53 does not bind directly to the IKKα promoter, overexpressed p53 interacts with ETS-1 to repress IKKα, resulting in repression of NF-κB signaling [33]. Ironically, activated p53 induces NF-κB DNA binding but suppresses its transcriptional activation through inhibition of IKK and histone H3 kinase on DNA [13]. In addition, p53 attenuates IKKβ activity by reducing glycolysis-linked protein O-glycosylation [14] [34]. In contrast, our findings revealed that p53 overexpression and stabilization reduced TNF-activated IκB (Figure 3C, 6B, S1B and S2) but had no effect on IKK expression (Fig. 5C, S1C and S2), suggesting that p53 is unlikely to affect IKK as an upstream target for phosphorylation of IκB in ovarian cancer cells.

Another mechanism through which p53 might regulate NF-κB signaling is by limiting the pool of coactivators or interacting with its components. Because p53 shares transcriptional coactivators p300/CBP with NF-κB signaling [15]–[17], p53 may inhibit p65-dependent transactivation by reducing the pool of p300/CBP. Overexpressed p300/CBP cannot completely restore the inhibitory effect of p65 on p53 activity and reversely p53 mutant cannot suppress p65 activity despite of its ability to bind p300/CBP [17]. In addition, IKKα phosphorylates CBP to enhance NF-κB signaling by switching the binding preference of CBP from p53 to NF-κB [35]. The p300/CBP is likely utilized according to dominantly activated signaling between p53 and p65. Because IκBα binds to p53 [27], p53 may bind to NF-κB components such as p65 and IκB followed by disruption of NF-κB signaling. Our finding that restoration of p53 had no significant effect on its physical binding to both p65 and IκB in ovarian cancer cells tested (Figure 5B) suggests that p53 binding to these components of NF-κB does not likely contribute to its inhibition of NF-κB activity.

Our observation that restoration of p53 increases accumulation of ubiquitinated proteins and reduces proteasome activity, resulting in increase of ubiquitinated IκB (Figure 4) suggests that proteasomal regulation is a major contributor to p53 regulation of NF-κB. It is well known that the proteolytic destruction of IκB by the ubiquitin-proteasome system plays a key role in the immediate elimination of IκB [36]. In addition, either wild type or mutant p53 can reduce ubiquitin-proteasome activity [37]. However, our examination of ubiquitin-related genes involved in p53-induced ubiquitination did not reveal any significant changes likely to contribute to increased ubiquitination (Table 1). Restoration of p53 specifically increased Mdm2 in all cell lines tested (Table 1 and Figure 5A). Many reviews document that Mdm2 is a p53 responsive gene and ubiquitinates p53 [38]–[39], so any effects of p53 on Mdm2 are self-limiting. Nonetheless, Mdm2 increases p65 promoter activity by reversing p53-mediated suppression of p65 in acute lymphoblastic leukemia cells [12], so it could contribute to p53's effects on NF-κB signaling via the ubiquitin-proteasome system.

In addition, immune cells such as neutrophils and macrophages lacking p53 have elevated responses to lipopolysaccharide (LPS) stimulation by producing more proinflammatory cytokines including TNF, IL-6 and CXCL2 via enhanced NF-κB activity [40]. Moreover, knockdown of p65 significantly induces protein levels of p53 in HepG2 human hepatocellular liver carcinoma cell line [41]. The p53-deficient condition can increase proinflammatory burden in the whole body as well as inflammation-associated cancers. Also aging is known to be associated with decreased function of p53, which enhances NF-κB signaling [30]. Because aging is a main risk factor for cancer, functional loss of p53 caused by aging could contribute to the increase in proinflammatory chemokines via enhanced NF-κB signaling.

**Figure 7:**
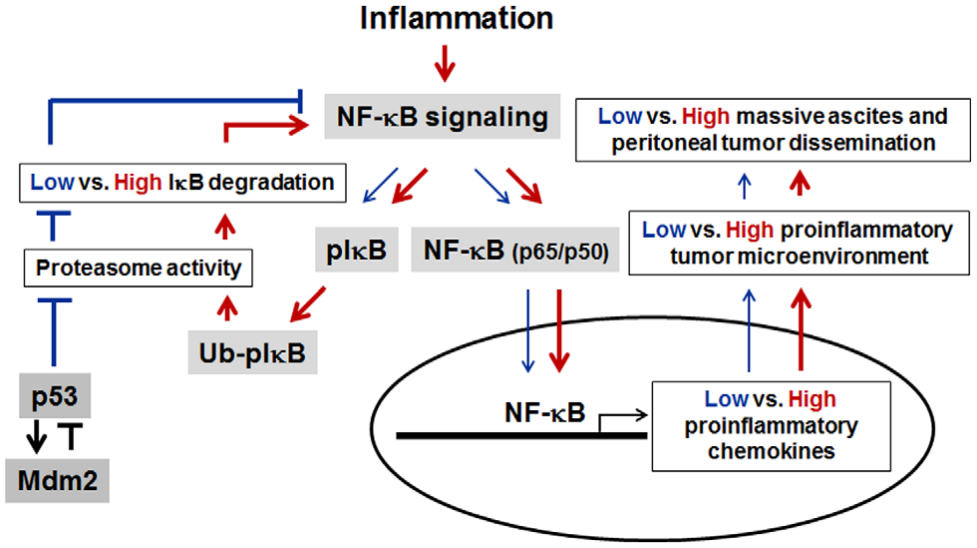
Schematic of the proposed mechanisms by which p53 expression regulates proinflammatory chemokines in ovarian cancer. Chronic inflammation promotes ovarian cancer progression via NF-κB signaling. Wild-type p53 reduces activity of the ubiquitin-proteasome system, resulting in low IκB degradation (blue line). This reduces NF-κB activity, inhibiting proinflammatory chemokine expression and attenuating the proinflammatory tumor microenvironment (blue arrow). On the other hand, p53 increases Mdm2 expression (dark arrow) in a feedback loop to compensate for the reduced activity of the ubiquitin-proteasome system. Loss of p53 observed frequently in advanced ovarian cancer triggers high proinflammatory chemokines by increasing NF-κB signaling which is composed of IκB and p65/p50 followed by a high IκB degradation (red arrow). Enhanced NF-κB activity results in potentiation of the proinflammatory tumor microenvironment for ovarian cancer progression such as peritoneal tumor dissemination and massive ascites. The imbalance between p53 and Mdm2 also contributes to increasing NF-κB signaling via the ubiquitin-proteasome system.

In conclusion, in ovarian cancer cells p53 attenuates expression of proinflammatory chemokines in response to inflammation, probably in part by blocking degradation of IκB via disrupted proteasome activity. This is possible because of the dominant imbalance of p53 in overwhelming Mdm2 (Figure 7).

## Supporting Information

Figure S1 **Characteristics of chemokine network and inhibitory effect of nutine-3 on TNF-activated IκB in p53 wild-type IGROV-1 ovarian cancer cells.** (A) Signature of chemokine ligands and receptors in IGROV-1 human ovarian cancer cells. After isolating total RNA, PCR array was performed using a customized PCR array plate containing complementary sequences for human chemokine genes. Different colors indicate average cycle threshold with expression ranges from >35 to <25. (B) Effect of nutlin-3 on TNF-activated IκB. (C) Effect of nutlin-3 on TNF-activated IKK. IGROV-1 cells were pretreated with nutlin-3 (10 µM) for 24 h followed by TNF (10 ng/ml) for indicated times. Whole cell lysates were prepared and Western blot was carried out using specific antibodies. β-actin serves as loading control. Experiments were performed in duplicate and a representative result is shown.
(TIF)

Figure S2 **Differential effect of nutine-3 on TNF-activated IκB between p53 wild-type and mutant ovarian cancer cells.** (A) Effect of nutlin-3 on TNF-activated IκB in p53 wild-type A2780 cells. (B) Effect of nutlin-3 on TNF-activated IκB in p53 mutant OVCAR-3 cells. Cells were pretreated with nutlin-3 (10 µM) for 24 h followed by TNF (10 ng/ml) for indicated times. Whole cell lysates were prepared and Western blot was carried out using specific antibodies. β-actin serves as loading control. Experiments were performed in duplicate and a representative result is shown.
(TIF)
