# Supplementary figures and images for: Inhibitory Effect of Tumor Suppressor p53 on Proinflammatory Chemokine Expression in Ovarian Cancer Cells by Reducing Proteasomal Degradation of IκB
Source: PLoS One. 2012 Dec 31;7(12):e51116. doi: 10.1371/journal.pone.0051116 (PMC3534106; doi:10.1371/journal.pone.0051116)

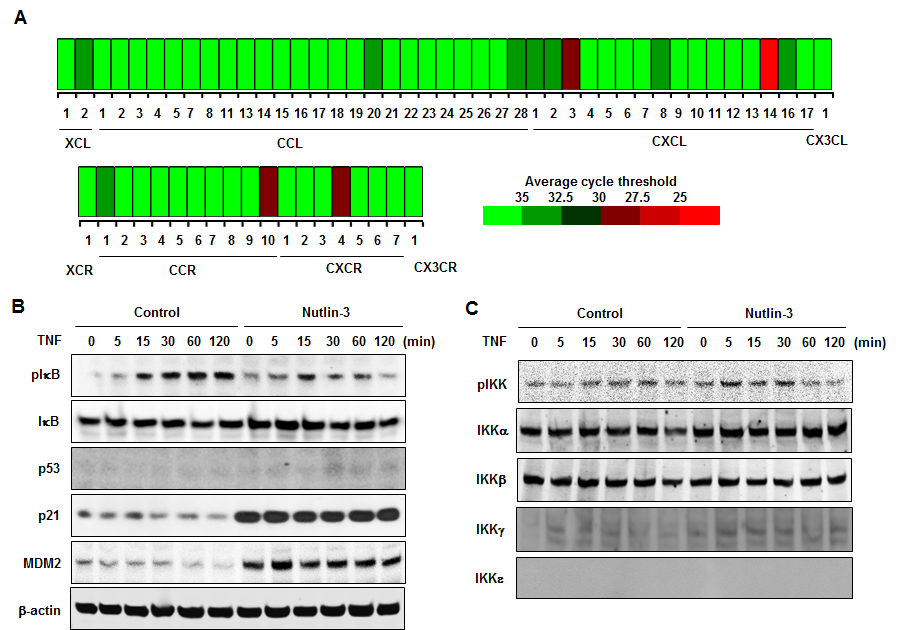

Supplement: Figure S1 — Characteristics of chemokine network and inhibitory effect of nutine-3 on TNF-activated IκB in p53 wild-type IGROV-1 ovarian cancer cells. (A) Signature of chemokine ligands and receptors in IGROV-1 human ovarian cancer cells. After isolating total RNA, PCR array was performed using a customized PCR array plate containing complementary sequences for human chemokine genes. Different colors indicate average cycle threshold with expression ranges from >35 to <25. (B) Effect of nutlin-3 on TNF-activated IκB. (C) Effect of nutlin-3 on TNF-activated IKK. IGROV-1 cells were pretreated with nutlin-3 (10 µM) for 24 h followed by TNF (10 ng/ml) for indicated times. Whole cell lysates were prepared and Western blot was carried out using specific antibodies. β-actin serves as loading control. Experiments were performed in duplicate and a representative result is shown. (TIF) [file pone.0051116.s001.tif]

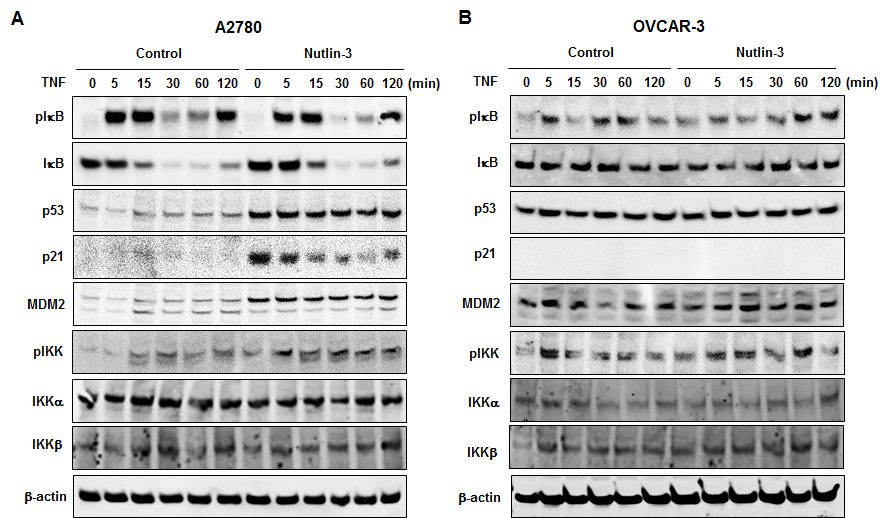

Supplement: Figure S2 — Differential effect of nutine-3 on TNF-activated IκB between p53 wild-type and mutant ovarian cancer cells. (A) Effect of nutlin-3 on TNF-activated IκB in p53 wild-type A2780 cells. (B) Effect of nutlin-3 on TNF-activated IκB in p53 mutant OVCAR-3 cells. Cells were pretreated with nutlin-3 (10 µM) for 24 h followed by TNF (10 ng/ml) for indicated times. Whole cell lysates were prepared and Western blot was carried out using specific antibodies. β-actin serves as loading control. Experiments were performed in duplicate and a representative result is shown. (TIF) [file pone.0051116.s002.tif]
